# Rare Association of Type 1 Omphalocele With Ileocecal Atresia and Congenital Appendiceal Agenesis: A Case Report

**DOI:** 10.7759/cureus.110746

**Published:** 2026-06-12

**Authors:** Mohamed Taha Mellouki, Zineb Benmassaoud, Yacine Zouirech, Hind Cherrabi

**Affiliations:** 1 Faculty of Medicine and Pharmacy, Ibn Zohr University, Agadir, MAR; 2 Department of Pediatric Visceral Surgery, Mother and Child Hospital, Mohammed VI University Hospital Center, Agadir, MAR

**Keywords:** appendiceal agenesis, ileocecal atresia, neonatal surgery, omphalocele, omphalocele minor, surgical case reports

## Abstract

Omphalocele is a relatively common congenital abdominal wall defect encountered in the neonatal period, whereas intestinal atresia and appendiceal developmental anomalies are much rarer entities. The association of these malformations represents an exceptionally rare clinical condition, scarcely reported in the literature.

We report a case of a female neonate presenting with a type 1 omphalocele associated with ileocecal atresia and congenital appendiceal agenesis. Surgical management included reduction of the herniated viscera, primary ileocolic anastomosis, omphalocele repair, and a prophylactic Ladd procedure. The postoperative course was uneventful, with early return of bowel function and progressive advancement of enteral feeding.

This case is reported due to the extreme rarity of this triad of congenital anomalies and to highlight key diagnostic and therapeutic considerations in neonatal surgical management. It also emphasizes the importance of early, tailored surgical intervention in achieving excellent functional outcomes.

## Introduction

Omphalocele remains one of the most frequently encountered congenital abdominal wall anomalies in the neonatal period, with an estimated incidence of 3.38 per 10,000 live births [1]. This malformation is characterized by protrusion of abdominal viscera through a defect in the umbilical ring, with the viscera remaining covered by an intact amniotic membrane. Contemporary classification distinguishes minor omphaloceles (type 1, < 5 cm) from giant omphaloceles (> 5 cm), with this distinction being critical for therapeutic planning [2]. Associated congenital anomalies in omphalocele are well-documented in recent literature, including cardiac malformations, chromosomal abnormalities, and gastrointestinal malformations [3]. However, the specific association between omphalocele and intestinal atresia remains exceptionally rare, in contrast to gastroschisis, where this association is observed in approximately 10% of cases [3]. Among intestinal atresias, involvement of the ileocecal region represents approximately 10-15% of cases, making it a less frequent location than proximal jejunal or ileal atresias [4]. Congenital appendiceal agenesis constitutes an extremely rare anomaly, with an incidence of less than 0.01% in historical autopsy series [5]. When identified, it is typically accompanied by other gastrointestinal malformations such as intestinal atresia, malrotation, or Meckel diverticulum [5]. The simultaneous presence of these three anomalies in a single patient appears to be exceptionally rare. Given the limited literature on this association and the lack of specific guidance regarding its surgical management, this case may contribute to a better understanding of its clinical and operative implications.

## Case presentation

A female neonate, delivered at term by spontaneous vaginal delivery with a birth weight of 3.2 kg, presented immediately after birth with a visible abdominal wall defect at the umbilical region. She was born at term by spontaneous vaginal delivery in a peripheral hospital. The mother was a 29-year-old primigravida with no significant medical history. She lived in a rural area and had not undergone regular prenatal follow-up; consequently, no antenatal ultrasound screening or fetal MRI had been performed, and the omphalocele was not diagnosed before birth. Physical examination revealed a type 1 omphalocele measuring approximately 4 cm in diameter, containing intestinal loops and covered by an intact amniotic membrane. The defect was immediately covered with sterile gauze soaked in 0.9% normal saline to prevent contamination and dehydration. Routine transthoracic echocardiography performed according to protocols for abdominal wall anomalies revealed no structural cardiac abnormalities. The infant’s general hemodynamic status was stable with no other apparent congenital malformations.

**Figure 1:**
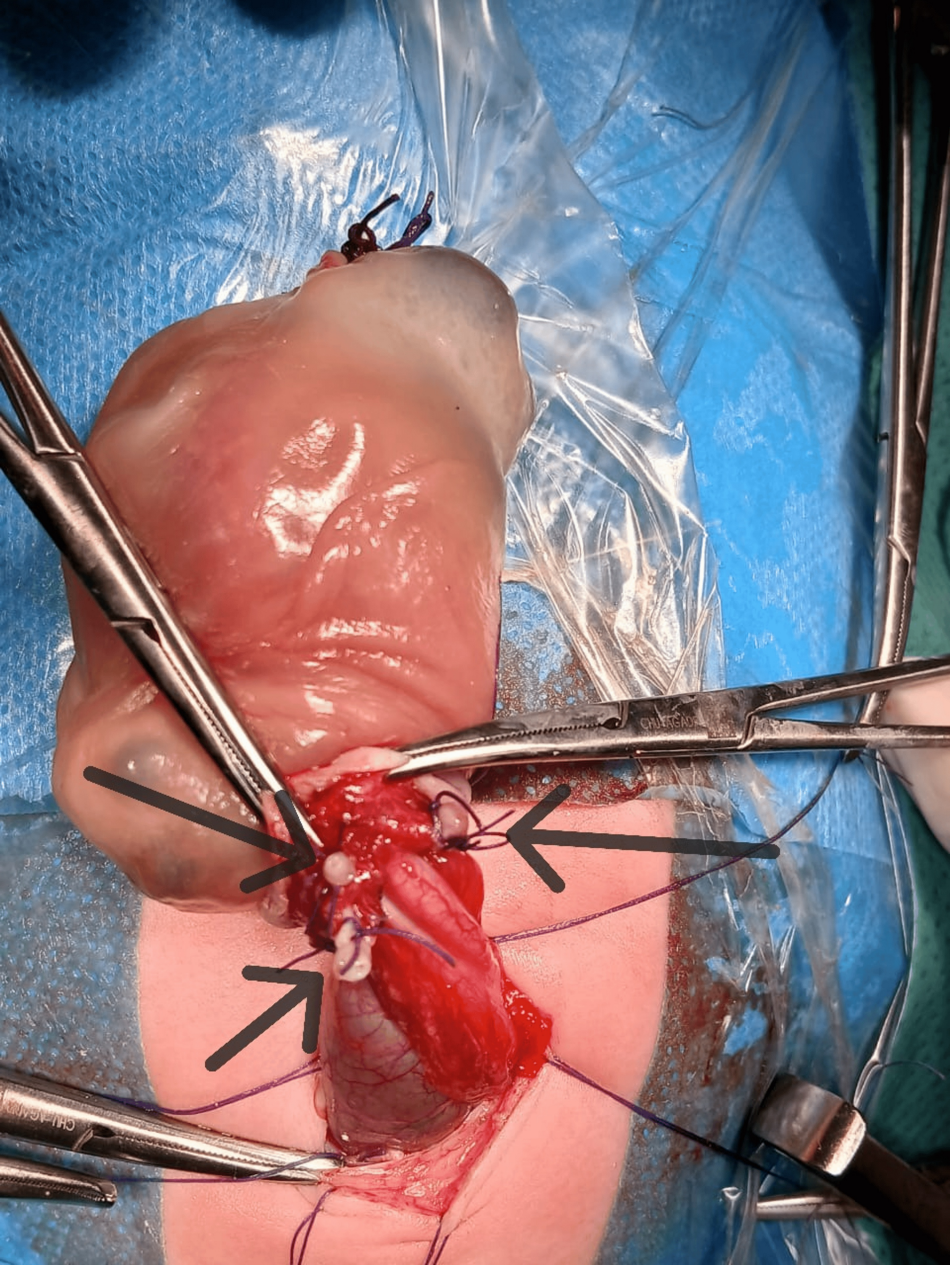
Intraoperative image showing a type 1 omphalocele with ligation of the two umbilical arteries and the umbilical vein (three arrows).

Following appropriate preoperative preparation, including informed parental consent and hemodynamic stabilization, the neonate was scheduled for surgical intervention on the day of birth. Under general anesthesia with endotracheal intubation, a circumferential incision at the umbilicus was made to access the omphalocele sac (Figure 1). The amniotic membrane was carefully excised, and intestinal loops were progressively reduced into the peritoneal cavity.

**Figure 2:**
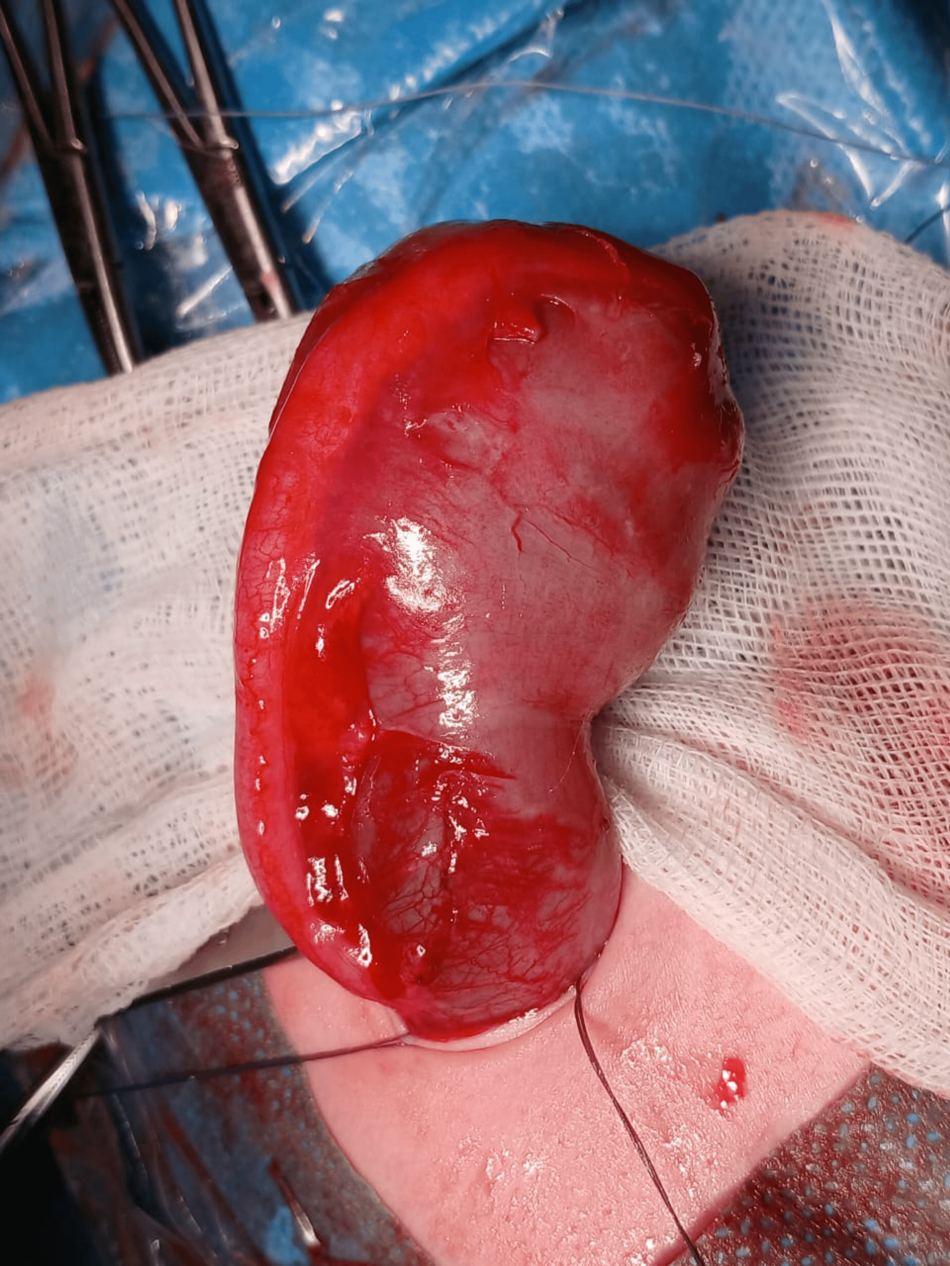
Intraoperative image showing ileal dilatation proximal to an atretic segment after removal of Wharton’s jelly.

Initial surgical exploration revealed a significantly dilated terminal ileal segment filled with meconium, located proximal to an area of atresia (Figure 2).

**Figure 3:**
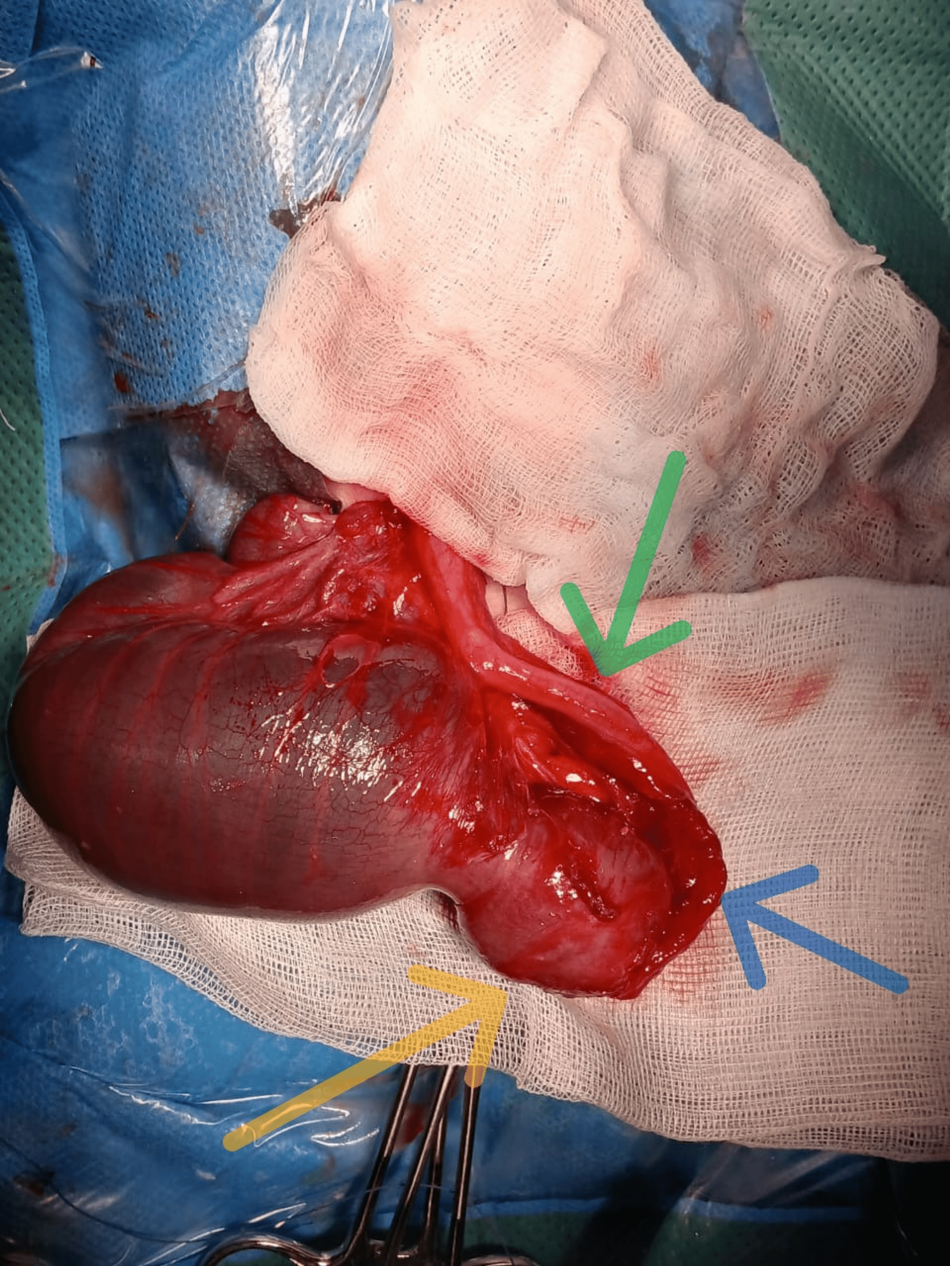
Intraoperative image showing ileocecal atresia. Yellow arrow: dilated terminal ileal loop. Blue arrow: ileocecal atresia with absent appendix. Green arrow: unused small-caliber ascending colon.

Careful dissection and systematic exploration of the entire gastrointestinal tract revealed complete atresia at the ileocecal junction, characterized by an abrupt transition between a dilated ileal segment and a collapsed ascending colon. Remarkably, careful examination of the ileocecal region revealed no appendiceal tissue (confirmed appendiceal agenesis) (Figure 3). Complete exploration of the remaining intestinal tract identified no other atresia sites. The intestines demonstrated normal rotation, excluding congenital malrotation.

**Figure 4:**
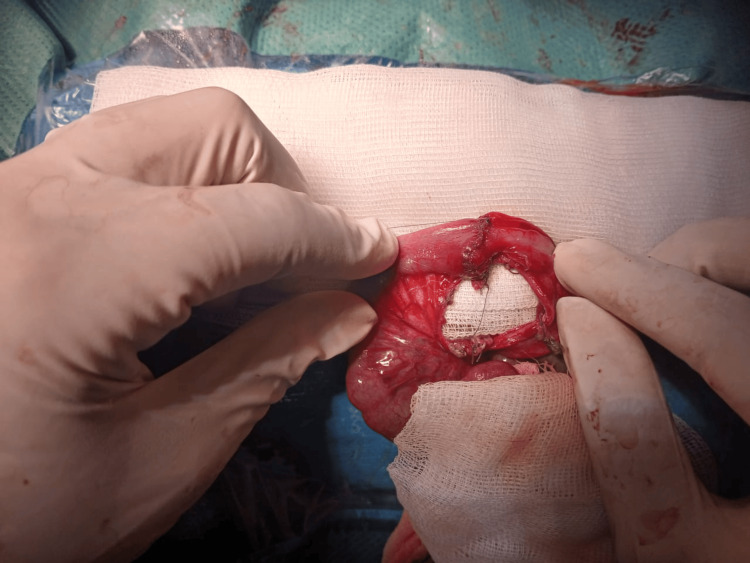
Intraoperative image showing a termino-terminal ileocolic anastomosis after remodeling plasty.

Due to the complexity of the intraoperative findings, the initial incision was extended to ensure adequate exposure and sufficient working space. The dilated ileal segment was minimally resected, preserving maximal intestinal length. An enlargement plasty was performed on the small-caliber segment to optimize its diameter and facilitate a tension-free anastomosis. A primary termino-terminal ileocolic anastomosis was then constructed using a continuous running suture with 5-0 absorbable Vicryl, ensuring good mucosal apposition and secure closure (Figure 4).

Although intraoperative examination confirmed normal intestinal rotation, a prophylactic Ladd procedure was performed at the discretion of the surgical team due to the complex associated congenital anomalies (omphalocele and ileocecal atresia) and concerns regarding potential abnormal intestinal fixation and future risk of volvulus. This procedure included repositioning of the ascending colon to the left abdomen, positioning of the small intestine to the right, systematic division of any potentially obstructive peritoneal bands (Ladd bands), and widening of the mesentery to reduce the risk of future volvulus.

Following completion of intestinal repair, the abdominal wall defect was closed in layers: fascial closure with interrupted 3-0 absorbable sutures, subcutaneous layer closure, and skin closure with interrupted 4-0 absorbable sutures. An intra-abdominal drain (10 French caliber) was placed in the peri-anastomotic position to allow early detection of postoperative complications. Total operative time was two hours and 15 minutes with minimal blood loss (<10 mL).

The neonate was transferred to the neonatal intensive care unit in a hemodynamically stable condition under mechanical ventilation and sedation, with continuous postoperative monitoring.

On postoperative day one, vital signs remained stable, and drain output was minimal, with a soft, mildly distended abdomen. On postoperative day two, passage of meconium was observed, allowing successful extubation, and trophic enteral feeding via nasogastric tube was initiated. On postoperative day three, ongoing passage of meconium confirmed anastomotic patency; the drain was removed, and feeding volumes were progressively increased. On postoperative day four, transition to oral feeding was initiated with breastfeeding, which was well tolerated, and abdominal examination remained normal. Between postoperative days five and seven, feeds were progressively advanced to full enteral nutrition (150 mL/kg/day) with excellent tolerance and appropriate weight gain. The patient was discharged on postoperative day seven in stable clinical condition without complications.

At the six-month follow-up, anthropometric evaluation revealed an excellent growth trajectory. Psychomotor development was appropriate for chronological age with normal gross and fine motor skills. Feeding was exclusively breastfed, and the stool pattern was normal. Abdominal examination showed a soft, non-distended abdomen with normal bowel sounds. The surgical incision was well-healed without a hernia. Routine laboratory tests were within normal limits. Parents were counseled on excellent recovery, the importance of continued vitamin supplementation, warning signs requiring medical attention, and long-term follow-up recommendations.

## Discussion

The association between omphalocele and intestinal atresia remains exceptional, with a prevalence of less than 1% of omphalocele cases [1]. This rarity contrasts sharply with gastroschisis, where intestinal atresia is observed in approximately 10% of cases [3]. The ileocecal region represents a less frequent location for intestinal atresia, accounting for approximately 10-15% of all intestinal atresias [4], making this case presentation particularly remarkable.

Congenital appendiceal agenesis constitutes an extremely rare anomaly. Although the precise embryological basis of this malformation remains incompletely elucidated, current data suggest it may result from failure of cecal diverticulum development during weeks seven to 12 of fetal gestation [5]. The coexistence of appendiceal agenesis with ileocecal atresia strongly suggests a common embryological insult affecting the ileocecal region during critical periods of fetal development. The simultaneous presence of these two anomalies with an omphalocele in a single patient is exceptionally rare, with only a handful of similar cases reported in the medical literature.

The etiology of intestinal atresia involves several proposed mechanisms. The intrauterine vascular accident theory, proposing vascular compromise or thrombosis of mesenteric vessels supplying the affected intestinal segment resulting in ischemic necrosis and subsequent atresia, remains the most widely accepted hypothesis [6]. Alternative mechanisms include abnormal meconium accumulation causing intestinal obstruction and secondary atresia [6]. Several genetic syndromes have been associated with intestinal atresia, including Feingold syndrome and familial intestinal atresia [7]. Maternal factors, including perinatal infections, exposure to certain medications, and nutritional deficiencies, have also been implicated in the pathogenesis of these malformations [8].

Surgical management of omphalocele with concomitant intestinal atresia requires meticulous planning and informed intraoperative decision-making. Surgical objectives include repair of the omphalocele defect with primary closure when technically feasible, correction of intestinal atresia with appropriate anastomosis, prevention of future complications such as malrotation and internal herniation, and minimization of postoperative morbidity through methodical and rigorous surgical technique [9].

In the present case, the decision to perform primary termino-terminal ileocolic anastomosis rather than a staged approach or diverting colostomy was justified by several factors: the infant was hemodynamically stable, the intestine was not severely contaminated, the anastomotic site was well-vascularized, and the surgical team possessed appropriate expertise in neonatal intestinal surgery. The use of absorbable sutures and the two-layer technique provided adequate mechanical strength and reduced the risk of anastomotic leak.

The prophylactic Ladd procedure, despite normal intestinal rotation, was justified by data indicating that intestinal atresia increases the risk of malrotation and internal herniation [10]. The Ladd procedure is a relatively simple procedure that can be performed without significant additional morbidity and may prevent future complications and reduce the need for subsequent operations.

Primary closure of the omphalocele defect was successful, avoiding the need for staged closure or prosthetic materials. This was possible because the defect was relatively small (type 1 omphalocele) and the abdominal cavity had adequate capacity to accommodate reduced viscera without excessive intra-abdominal pressure increase.

The postoperative course was remarkably favorable. Early meconium passage on postoperative day two constituted a favorable clinical indicator of successful anastomotic function. Early extubation and rapid initiation of enteral feeding contributed to quick recovery. The use of an intra-abdominal drain allowed early detection of any anastomotic leak or complication. Minimal drain output and early drain removal indicated well-healed anastomosis without significant complications.

Early enteral feeding, even in small amounts (trophic feeding), has been demonstrated to promote intestinal adaptation and improve outcomes in neonates undergoing intestinal surgery [10]. Progressive advancement of feeding volumes and transition to full enteral feeding by postoperative day seven provided appropriate nutritional support without overwhelming the newly created anastomosis.

The simultaneous presence of type 1 omphalocele, ileocecal atresia, and congenital appendiceal agenesis in a single patient represents an exceptionally rare clinical entity. Medical literature reveals only a handful of cases describing the association of omphalocele and intestinal atresia, and even fewer describing concomitant appendiceal agenesis [11]. This case significantly adds to the limited body of literature on this rare combination of anomalies and demonstrates successful surgical management of a complex congenital condition. The favorable short-term outcome at six months, with normal growth, appropriate psychomotor development, and optimal gastrointestinal function, is encouraging. However, long-term multidisciplinary follow-up is essential to monitor for potential late complications.

Neonates with intestinal atresia and primary anastomosis remain at risk for several long-term complications. Postoperative adhesions constitute a common complication of neonatal abdominal surgery and may result in intestinal obstruction in the future, with a reported incidence of 5-15% following intestinal atresia repair [11]. Anastomotic site stenosis may develop months or years after initial surgery, being more frequent when significant size discordance exists between proximal and distal intestinal segments. In the present case, tapering plasty of the dilated ileal segment reduced the risk of anastomotic stenosis. Neonates undergoing intestinal surgery carry an increased risk of malabsorption of fat-soluble vitamins (A, D, E, K) and other essential nutrients, justifying prolonged supplementation [11]. The congenital absence of the appendix in this patient completely eliminates the risk of appendicitis, although it may theoretically be associated with other gastrointestinal anomalies, warranting vigilant long-term follow-up [12].

## Conclusions

This case describes a rare association of type 1 omphalocele, ileocecal atresia, and congenital appendiceal agenesis in a neonate, highlighting the complexity of gastrointestinal anomalies that may be encountered in patients with abdominal wall defects. It underscores the importance of meticulous and systematic intraoperative exploration to detect associated abnormalities that may not be identified preoperatively and that can directly influence surgical management. In selected cases with favorable clinical and local conditions, primary ileocolic anastomosis can be safely performed as a single-stage procedure. The indication for prophylactic Ladd procedure in the setting of normal intestinal rotation remains controversial and should be individualized based on intraoperative findings and surgical judgment. Although the short-term outcome was favorable, longer follow-up is necessary to assess long-term functional results and potential late complications.
